# Why Ablation of Sites With Purkinje Activation Is Antiarrhythmic: The Interplay Between Fast Activation and Arrhythmogenesis

**DOI:** 10.3389/fphys.2021.648396

**Published:** 2021-03-23

**Authors:** Ruben Coronel, Mark Potse, Michel Haïssaguerre, Nicolas Derval, Mathilde R. Rivaud, Veronique M. F. Meijborg, Matthijs Cluitmans, Mélèze Hocini, Bastiaan J. Boukens

**Affiliations:** ^1^Department of Experimental Cardiology, Amsterdam Cardiovascular Sciences, Amsterdam UMC, University of Amsterdam, Amsterdam, Netherlands; ^2^IHU Liryc, Electrophysiology and Heart Modeling Institute, Fondation Bordeaux Université, Bordeaux, France; ^3^UMR5251 Institut de mathématiques de Bordeaux, Talence, France; ^4^Carmen Team, Inria Bordeaux ‐ Sud-Ouest, Talence, France; ^5^Department of Medical Biology, Amsterdam Cardiovascular Sciences, Amsterdam UMC, University of Amsterdam, Amsterdam, Netherlands

**Keywords:** idiopathic ventricular fibrillation, arrhythmias, early repolarization syndrome, Purkinje, ablation, electrophysiology

## Abstract

Ablation of sites showing Purkinje activity is antiarrhythmic in some patients with idiopathic ventricular fibrillation (iVF). The mechanism for the therapeutic success of ablation is not fully understood. We propose that deeper penetrance of the Purkinje network allows faster activation of the ventricles and is proarrhythmic in the presence of steep repolarization gradients. Reduction of Purkinje penetrance, or its indirect reducing effect on apparent propagation velocity may be a therapeutic target in patients with iVF.

## Introduction

Patients who have survived idiopathic ventricular fibrillation (iVF) are typically difficult to treat and often rely on an implanted Cardioverter Defibrillator (ICD) to restore normal heart rhythm (Patton et al., 2016). Single ventricular premature beats that trigger iVF may originate in Purkinje cells (Gianni et al., 2018). Accordingly, removing the trigger by ablation of regions showing Purkinje activity reduces recurrence of ventricular fibrillation in the majority of patients (Haissaguerre et al., 2002; Bänsch et al., 2003; Bogun et al., 2006). The treatment, however, is intriguing because “pruning” of sites with Purkinje spikes around the target area adds to the success (Cheniti et al., 2018; Haïssaguerre et al., 2020). Therefore, we provide an alternative hypothesis stating that ablation of Purkinje fibers prevents arrhythmia reoccurrence by reducing the amount of Purkinje tissue and not, or not only, by removing the trigger.

### Variability Exists in the Extent of the Purkinje System

The cardiac Purkinje network is highly variable between species and within species, especially in the extent of the peripheral branches (Truex and Smythe, 1965; Demoulin and Kulbertus, 1972; Ono et al., 2009; Sedmera and Gourdie, 2014; Anderson et al., 2017). The ventricular conduction system develops from the embryonic trabeculae (Christoffels and Moorman, 2009; Jensen et al., 2012). During development, part of the trabeculae will become Purkinje cells whereas the remainder acquires a working myocardial phenotype. The primordial Purkinje trabeculae require further specialization after birth under influence of Nkx2-5 and Irx3 expression and Notch signaling (Miquerol et al., 2004; Meysen et al., 2007; Zhang et al., 2011). Individual variability in expression of these factors influences penetration of the Purkinje network into the ventricular wall. This is illustrated in mice with over-activation of Notch signaling, where the Purkinje cells can be found deeper in the ventricular wall (Rentschler et al., 2012). The observation that the precursors of the cardiac conduction system are present along the entire human embryonic endocardium (Christoffels and Moorman, 2009) whereas it is merely present in a loose network in the adult heart suggests that the extensive form of a Purkinje system is disadvantageous. We speculate that natural selection has limited the Purkinje system to an optimal size, allowing synchronized myocardial contraction without the risk of reentrant arrhythmias. Rapid activation is thus matched with the physiological repolarization gradients. Arrhythmias may develop if the repolarization gradients become steeper in the course of life or as a result of pathological processes. Natural selection is no longer operative in that stage of life.

### Deeper Penetration of Purkinje Fibers Shortens QRS_50_ But Not QRS_100_

**Figure 1:**
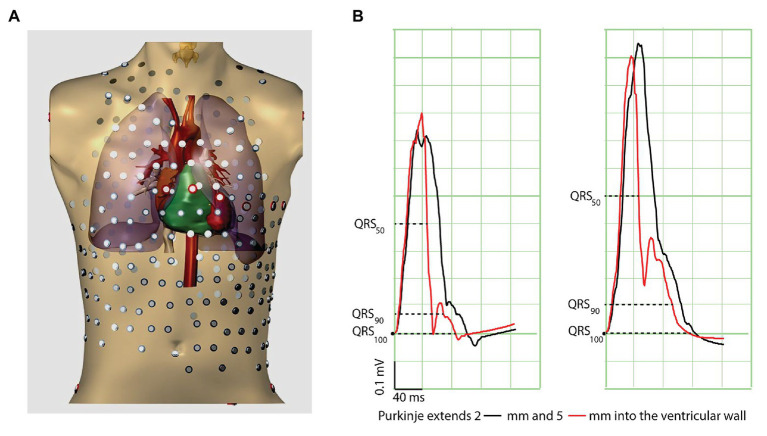
Computer simulations show that penetration of Purkinje fibers into the ventricular wall results in QRS slurring and short QRS50. **(A)** Torso model used for simulation. **(B)** The traces show simulated electrocardiograms (Lead II and III) with Purkinje fibers penetrating 2 mm (black) and 5 mm (red) into the ventricular wall. The dashed lines refer to the QRS duration at 50, 90, and 100% at 5 mm penetrance into the ventricular wall. Note that these moments of QRS50, QRS90, and QRS100 slightly differ at 2 mm penetrance because of different QRS amplitudes.

Because the Purkinje system transfers activation to the working myocardium, and because the latter has slower conduction properties than Purkinje tissue, the ventricular activation process can be conceived as consisting of two components, one (in Purkinje-rich tissue) predominantly through Purkinje-muscle conduction, and the other (in Purkinje-void tissue) predominantly through muscle-muscle conduction. Thus, the end of the QRS complex is generated by muscle-muscle conduction only. In the presence of a more penetrating Purkinje network, a relatively larger contribution of (fast) Purkinje-mediated conduction is expected at the beginning of the QRS complex, whereas the end of the QRS complex is relatively unaffected. We therefore reasoned that a Purkinje system that extends slightly more into the walls of the ventricles leads to a shorter QRS “body” (QRS_50_, QRS duration at 50% of the QRS amplitude). We tested this in a computer model of a human heart and torso (Figure 1A).

Propagating action potentials were simulated with a whole-heart reaction-diffusion model at 200 μm resolution (Hoogendijk et al., 2011; Potse, 2018). Membrane ionic currents were computed with a human ventricular membrane model that included the differential characteristics of subendocardial, midmyocardial, and subepicardial myocytes (ten Tusscher et al., 2004). Transmural fiber rotation was represented in the model. The ECG was computed using a bidomain model of the human heart and torso, including lungs and intracavitary blood volumes (Potse, 2018). The model incorporated a Purkinje network that penetrated into the ventricular walls. The degree of penetration was varied from 2 mm (from the endocardium into the wall) to 5 mm. Purkinje cells were modeled by incorporating a larger surface to volume ratio (leading to a 2× larger conduction velocity than normal). Simulations were performed on a Bullx cluster computer.

The black traces in Figure 1B represent the control condition where the Purkinje-system extended 2 mm into the left ventricular wall. When the Purkinje network extended 5 mm into the left ventricular wall, the QRS complex became shorter especially at mid-amplitude and was followed by J-waves similar to those in patients with the Early Repolarization pattern. The end of the QRS complex was less affected (Supplementary Table S1). Thus, our simulations indicate that a deeper penetrating Purkinje network is associated with a short QRS_50_ and a J-wave (ER pattern) as an expression of relatively late activated tissue. This generates the question whether humans with a short QRS_50_ are more at risk for idiopathic ventricular re-entrant arrhythmias (Wolpert et al., 2008).

## How Deeper Penetrated Purkinje Fibers Facilitate Unidirectional Block

**Figure 2:**
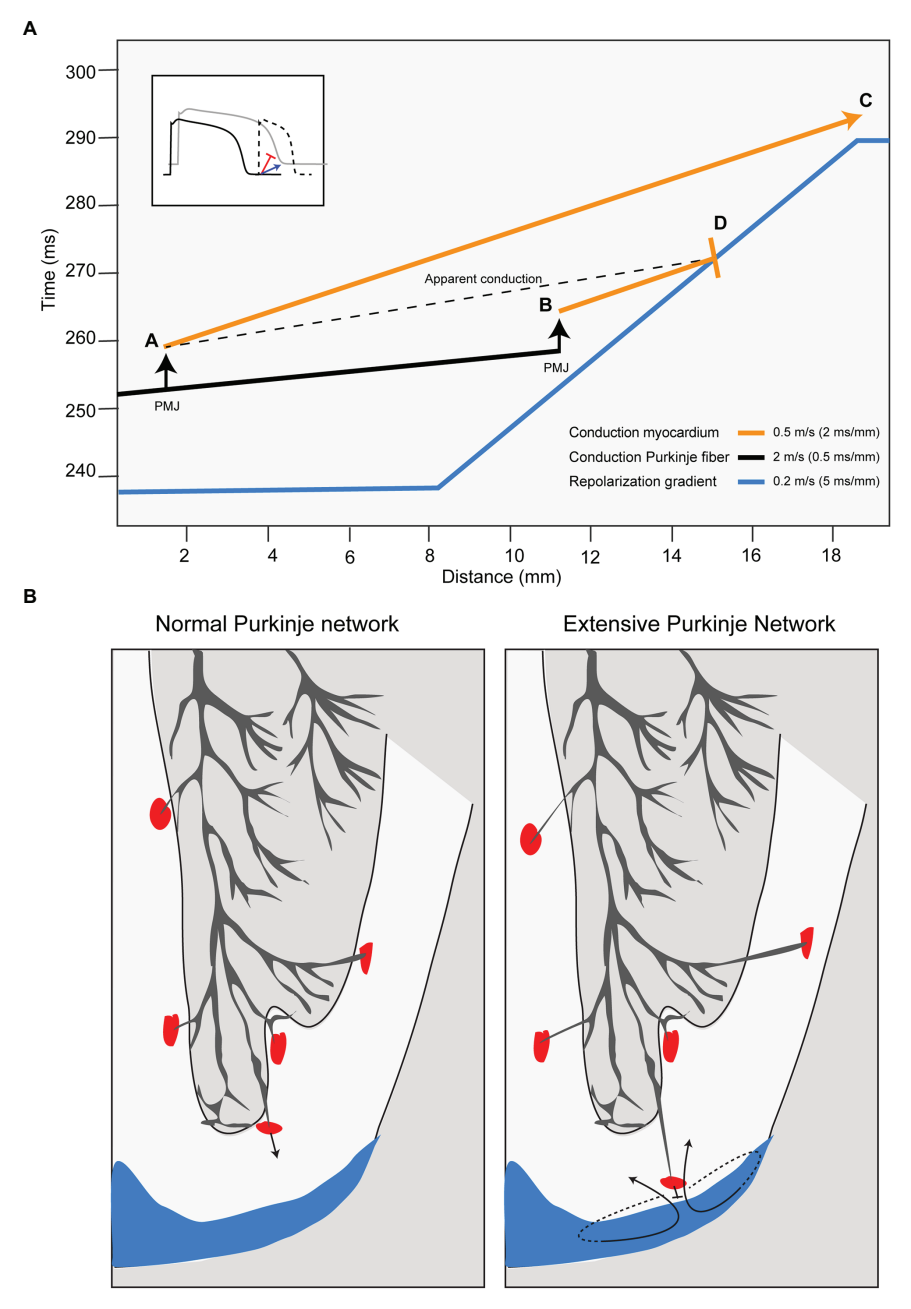
Purkinje-muscle junctions (PMJs) close to repolarization gradients may cause reentrant arrhythmias. **(A)** The graph shows time (vertical axis) and myocardial distances (horizontal axis). The blue line represents the repolarization time along the myocardial axis of beat 1 (supposedly a sinus activation). The orange and black lines show activation time along the myocardial axis of beat 2 (a ventricular premature activation). The orange line represents conduction through working myocardium and the black line through Purkinje fibers. If the PMJ activates the working myocardium far from the repolarization gradient (location A) the activation front propagates at low speed toward repolarization gradient, which will have disappeared when the activation front arrives (location C). If the PMJ activates the working myocardium close to the repolarization front (location B), the activation front meets still depolarized myocardium and conduction will block (location D) setting the stage for re-entry. Inset: A slowly conducting premature activation front (blue) arrives late at the site of prolonged repolarization causing activation whereas a fast conducting activation front (red) results in unidirectional block and re-entry. PMJ, Purkinje-muscle junction. **(B)** In the normal human heart, the Purkinje system is located at the endocardial side of the ventricular myocardium (left panel). If the Purkinje system penetrates into the transmural wall of the ventricle, the change is higher the working myocardium is activated close to depolarized myocardium (blue) and conduction block occurs.

Fast ventricular activation is a typical characteristic of the Purkinje system, especially if it penetrates deeper into the ventricular wall or if it is more extensive. This is schematically demonstrated in Figure 2A. The blue line in Figure 2A shows a local repolarization gradient (induced by a sinus beat) over a distance of myocardium (horizontal axis). Its steepness (s/m) conforms to recent repolarization mapping studies in normal human hearts (Opthof et al., 2017). The figure also shows the activation time (vertical axis) of a premature beat propagating along a Purkinje fiber (black line) from a point located at the far left. Its slope represents the reciprocal of conduction velocity (s/m, a steeper slope indicates a slower conduction). Conduction velocity in the Purkinje network is high (in this example 2 m/s) compared to that of working myocardium (here 0.5 m/s; Hoffman et al., 1959). At the sites indicated by the arrows, the activation wave transfers from Purkinje conduction to conduction along the working myocardium (orange lines) at the Purkinje-muscle junction (PMJ). The “apparent” conduction velocity AD is faster than the conduction velocity along myocardium AC. Thus, the risk of conduction block is increased if the PMJ is closer to the foot of the repolarization gradient causing the apparent conduction velocity to be faster than the reciprocal of the slope of the repolarization gradient. Figure 2B shows the concept in a cross section of the heart with a normal distribution of Purkinje fibers (Haissaguerre et al., 2016) and with a more extensive distribution of Purkinje fibers. The blue areas mark regions with a prolonged repolarization. The figure suggests that rapid conduction along a Purkinje fiber facilitates unidirectional block and reentry, and that ablation of the Purkinje fibers proximal to the repolarization gradient is antiarrhythmic.

## Discussion

Here, we propose that rapid activation of the heart due to an extensive Purkinje system in the presence of steep repolarization gradients promotes unidirectional block and subsequent reentry. Our hypothesis appears to be contradicting the insight that conduction slowing is arrhythmogenic. However, our hypothesis defines the first occurrence of (unidirectional) block and not the maintenance of the arrhythmia.

We have demonstrated earlier that conduction may modulate the arrhythmogenic substrate formed by a repolarization heterogeneity and may either promote or suppress the induction of reentry by a premature beat depending on the site of application of sodium channel blockade relative to a repolarization gradient (Coronel et al., 2010). Conduction slowing in the myocardial region with the shorter repolarization is antiarrhythmic (Coronel et al., 2010). We here reasoned that the opposite is also valid and that local conduction speeding in that region is pro-arrhythmic. In general, conduction is safe when it is slower than the reciprocal of the maximum repolarization gradient. Conversely, if repolarization gradients develop that are steeper than the reciprocal of the apparent conduction velocity (for example, in the course of life, or resulting from diseases like the long QT syndrome), reentrant arrhythmias are more likely to follow a premature activation. From Figure 2A, it can be derived that when the apparent conduction is faster, a larger range of coupling intervals (and a larger area from which premature beats originate) exists at which a premature beat causes re-entry (a larger vulnerable window).

Earlier studies have shown that fast activation of the heart, and shortening of the QRS duration, relates to the extensiveness of the Purkinje system (Goldberger et al., 1985; Berger et al., 1993; Allison et al., 2007). Our computer simulation, however, indicates that when deeper penetration of the Purkinje system into the ventricular wall only activates part of the myocardium earlier, it may leave some areas being activated relatively late, which can give rise to notching and slurring at the end of the QRS complex (Figure 1B; Meijborg et al., 2016). This is in line with our recent view on the mechanisms underlying the early repolarization pattern (Boukens et al., 2019, 2020; Haïssaguerre et al., 2019). The computer model was not tested for arrhythmogenesis.

Whether the Purkinje system indeed penetrates deeper into the ventricular wall of patients with iVF is yet to be investigated. Evidence of the involvement of the Purkinje system with iVF was provided by Koizumi et al. (2016) who discovered two novel mutations in the gene encoding IRX3, a transcription factor involved in the development and electrophysiological characteristics of the Purkinje system, in family members of patients with iVF. They also investigated mice deficient for Irx3 and found higher incidence of premature beats and sustained ventricular arrhythmias than in control mice. Their observations indicate that these arrhythmias result from altered expression of ion channels within the Purkinje system facilitating the spontaneous generation of action potentials. Whether the Purkinje network extended into the ventricular walls of these mice was not studied.

Indirect evidence of the involvement of the Purkinje system in iVF comes from the observation that Purkinje spikes often precede premature beats that give rise to ventricular fibrillation in iVF patients. Ablation of sites showing Purkinje spikes is often antiarrhythmic (Haissaguerre et al., 2002). It is thought that by ablation of sites with Purkinje spikes the PMJ, and thereby the source of the arrhythmias, is destroyed. However, local potentials showing Purkinje spikes do often also show ventricular complexes with a prominent R wave which, maybe more importantly, occur up to 100 ms after the Purkinje spike. This indicates that the ventricular myocardium activates elsewhere after which the activation front propagates toward the recording electrode giving rise to the R wave. Ablating regions showing these Purkinje spikes will reduce the extent of Purkinje network and reduces the apparent conduction velocity (the ratio of the conduction time and distance between A and C in Figure 2) and is thereby antiarrhythmic.

It cannot be excluded that ablation of sites showing Purkinje spikes reduces the fine network of the Purkinje fibers, which, in turn, reduces the anatomical substrate for reentry. The Purkinje network as substrate for reentry was already recognized by Schmitt and Erlanger (1928) who have provided a model for the mechanism of Purkinje-muscle reentry that has served as a concept for the understanding of anatomical re-entry in general. It is based on a Purkinje fiber that branches into two fibers both connecting working myocardium. The triangular organization facilitates reentrant activation. This concept has been applied to the mechanism of bundle branch re-entry, atrioventricular nodal reentry tachycardia and atrioventricular reentry tachycardia. Waxman et al. (1980) have described a similar mechanism but within a Purkinje fiber. Purkinje fibers potentially also play a role in the genesis of unidirectional block. Heterogeneity in the moment of recovery from inexcitability (or of full repolarization) is an important factor for the genesis of arrhythmias as it can lead to unidirectional block following a premature beat (Rivaud et al., 2021). Purkinje fibers typically have a longer action potential than the surrounding working myocardium (Zaza et al., 1989). Purkinje fibers are electrically isolated from the surrounding myocardium except at the PMJs. Therefore, differences in action potential duration are maintained even in peripheral branches of the Purkinje system where the action potential may even be longer than in the bundle branches (Myerburg et al., 1970). These areas with long local action potential duration are referred to as gates (Myerburg et al., 1970) and can cause conduction block leading to reentry when an ectopic beat originates nearby (Myerburg, 1971). These gates have been found by Walton et al. (2014) as well using optical mapping.

## Conclusion

Although the above ideas are speculative and not substantiated by systematic studies, our findings suggest that cardiac conduction velocity has a safe upper limit in the presence of pre-existing repolarization heterogeneities. Moreover, the reduction of apparent propagation velocity by ablating parts of the Purkinje system may underlie the antiarrhythmic efficacy of this therapeutic approach. These contentions need to be tested clinically.

## Data Availability Statement

The raw data supporting the conclusions of this article will be made available by the authors, without undue reservation.

## Author Contributions

RC and BB designed and wrote the manuscript. MP performed computer simulation and edited the manuscript. MHa, ND, MR, VM, MC, and MHo edited the manuscript. All authors contributed to the article and approved the submitted version.

### Conflict of Interest

MC is part-time employed by Philips Research.

The remaining authors declare that the research was conducted in the absence of any commercial or financial relationships that could be construed as a potential conflict of interest.
